# High-performance hydrogen gas sensor based on Ag-incorporated ZnO nanoparticles

**DOI:** 10.1038/s41598-025-22222-9

**Published:** 2025-11-03

**Authors:** Reza Torkamani, Bagher Aslibeiki, Saeid Salari, Hamid Azizi, Davide Peddis, Tapati Sarkar

**Affiliations:** 1 Faculty of Physics, University of Tabriz, Tabriz, Iran; 2 Department of Materials Science and Engineering, Uppsala University, Box 35, 75103 Uppsala, Sweden; 3 Department of Physics, Isfahan University of Technology, Isfahan, 84156-83111 Iran; 4 Institute of Physics, RCQI, Slovak Academy of Sciences, Dúbravská Cesta 9, 84511 Bratislava, Slovakia; 5 Department of Chemistry and Industrial Chemistry & Genova, INSTM RU, nM2-Lab, University of Genova, 16146 Genova, Italy; 6 Institute of Structure of Matter, nM2-Lab, National Research Council, Via Salaria Km 29.300, 00015 Roma, Monterotondo Scalo Italy

**Keywords:** Zinc oxide, Sliver-incorporation, Gas sensor, Surface metallization, Environmental monitoring, Semiconductors, Nanoparticles, Composites, Sensors, Sensors and biosensors

## Abstract

ZnO-based sensors often suffer from low response rates and long response and recovery times. To address this issue, Ag-incorporated ZnO-based gas sensors with Ag contents of 2, 4, 6, and 8% were synthesized using a simple, fast, and cost-effective method, making them promising candidates for future industrial applications. Structural analysis confirmed the presence of Zn–O bonding and the incorporation of silver as a secondary metallic phase, well integrated with the ZnO nanoparticles. Gas sensing tests were performed under different conditions, and the sample with 2% Ag content exhibited an extraordinary response of 4357%, approximately 29 times higher than that of pure ZnO nanoparticles. The sample with 8% Ag content showed the lowest response and recovery times. Additionally, the response rates of the samples were positively correlated with both concentration and temperature. The increase in response rate was attributed to the spill-over effect in the samples, which enhanced hydrogen mobility. The results demonstrated that Ag-doped ZnO nanoparticles exhibited higher porosity compared to the pure ZnO sample. This suggests that tuning the porosity or structure can further enhance the performance of ZnO-based sensors. Moreover, the fabricated sensor showed high sensitivity and an exceptionally low detection limit, indicating strong potential for the continued development of ZnO nanostructure-based gas sensors.

**Supplementary Information:**

The online version contains supplementary material available at 10.1038/s41598-025-22222-9.

## Introduction

Hydrogen (H_2_) is an extremely useful gas, a renewable source of energy, alternative fuel, clean and sustainable that has great potential for future application^1–3^. It is a non-poisonous gas that is lightweight compared to air; however, it can cause suffocation at high concentrations. The lower explosive limit of hydrogen in air is 41,000 parts per million (ppm) and at 10% of this amount, evacuation is recommended^4^. Due to its reactivity and hazardousness, it is essential to develop efficient methods for H_2_ detection^2,5^. Moreover, hydrogen gas is colorless and odorless, which makes its early detection more difficult^6,7^. With the increasing use of hydrogen, the necessity for developing new electronic devices that can detect hazardous gases has thus increased. Various materials have been applied for H_2_ gas sensing. For example, Askar et al. developed a H_2_ gas sensor based on nanostructured polyaniline with a detection limit as low as 1 ppm. However, the response and recovery times of their sensors were relatively high^8^. Semiconductor oxide-based multifunctional devices are one of the most relevant types for this purpose. In this context, new functional semiconductor oxide-based devices are being increasingly investigated due to their improved safety, good sensing response, and increased efficiency^9,10^. In a study on CeO_2_–SnO_2_ mixed oxide heterostructures, response and recovery times of 17 and 24 s, respectively, were observed for 40 ppm H_2_ gas. However, these values are relatively high and need to be reduced for practical applications^11^. Gas sensors with fast response, improved selectivity, and good stability are thus still lacking.

Among different gas sensors based on semiconductors, zinc oxide (ZnO) with its direct and wide bandgap, good carrier mobility, high shelf-life, intrinsic defects, good stability, and high exciton binding energy is suitable for the field of gas sensors^12^. It is used extensively in gas sensors^13^, solar cells^14^, photocatalysts^15^, supercapacitors^16^, and many other applications. Although metal oxide nanostructures are preferred due to their low-cost production and proper gas characterization, it is necessary to improve their response, response time, and recovery time. For this purpose, the performance of gas sensors based on semiconductors has been improved using strategies such as doping^17,18^, decoration^19^, increasing the porosity^20^, or some of their combinations^21,22^. In addition, the production methods of different nanostructures and the control of their synthesis parameters play an important role in the efficiency of the gas sensor. ZnO nanostructures such as nanorods^23^, nanoparticles (NPs)^24^, and thin films^25^ can be prepared using methods such as hydrothermal^23,26^, sol-gel^27^, spin-coating^28^, and chemical bath deposition^25^. ZnO-based composites have gained significant attention in recent years. Recently a Pd-doped rGO/ZnO–SnO_2_ sensor, which exhibited excellent response and recovery times (4 and 8 s, respectively) at 100 ppm of H_2_ gas, has bene developed. However, the synthesis of this composite is complex and costly, and the fabrication process is time-consuming. Therefore, more suitable sensing conditions can potentially be achieved using simpler and more cost-effective structures^29^.

As mentioned above, gas sensors have limitations such as low response, high response/recovery time, instability, and high working temperature. Continuous efforts are therefore being made to overcome these challenges. Agarwal et al.^30^ modified ZnO NPs with Ag NPs. The Ag-ZnO sensors achieved the highest value of response of ~ 480 at 300 ppm H_2_ exposure and also showed response and recovery times of 175 and 655 s, respectively, for hydrogen gas. Acharya et al.^25^ fabricated ZnO thin films to detect formaldehyde gas. The maximum response was 17.8 at 400 ppm of formaldehyde gas and the response and recovery times were 25 and 58 s, respectively. Yang et al.^31^ prepared SnO_2_ thin film, which exhibited the highest response value (378%) at 50,000 ppm and the response and recovery times for H_2_ gas sensing were 12 and 53 s, respectively. To evaluate the repeatability of the sensor, 8 testing cycles were carried out at 300 °C and it was observed that the sensor demonstrates excellent repeatability, maintaining nearly the same response for 8 successive sensing tests when switching between hydrogen and air. Regmi et al.^32^ reported suspended graphene (Gr)/poly (3,4-ethylene dioxythiophene):poly (styrene sulfonate)—polyethylene oxide composite nanoscale channels for H_2_ gas sensing. The sensing response was 6% at 100 ppm and 100 °C and the response and recovery times were 240 and 16 s, respectively. In general, previous research shows that pure ZnO nanostructures exhibit low sensitivity and relatively long response and recovery times. On the other hand, the development of complex composite structures often involves high costs and time-consuming fabrication processes^33^.

Compared to the reported results of gas sensors with low response and high response/recovery times, in this work, we have prepared ZnO NPs by thermal decomposition^34^ as a low-cost method, and notably, increased their gas sensor response to an extraordinarily high value of ~ 4360%, while at the same time reducing the response and recovery times to 4.3 and 6.5 s, respectively.

## Experimental section

### Preparing ZnO NPs

Zinc acetate dihydrate (Zn(CH_3_COO)_2_·2H_2_O) and citric acid (C_6_H_8_O_7_) were purchased from Merck company; silver nitrate (AgNO_3_) was purchased from NEUTRON company.

In this work, ZnO NPs were synthesized via a thermal decomposition method, known for its simplicity, cost-effectiveness and suitability for large-scale production. Zinc acetate dihydrate (Zn(CH_3_COO)_2_·2H_2_O) and citric acid (C_6_H_8_O_7_) were used as the zinc precursor and complexing agent, respectively, in a molar ratio of 1:1. For doping, silver nitrate (AgNO_3_) was added to the mixture in different molar percentages (0, 2, 4, 6, and 8%) relative to the amount of zinc acetate.

The calculated amounts of zinc acetate dihydrate, citric acid, and silver nitrate (as applicable) were accurately weighed using an analytical balance and then thoroughly mixed and ground using an agate mortar and pestle for approximately 30 min to ensure homogeneity. The resulting powder mixture was transferred to an alumina crucible and placed in a furnace.

The thermal decomposition process was carried out by heating the samples at 600 °C for 3 h in air. During this process, the organic components decomposed and pure and Ag-doped ZnO NPs were formed. After cooling to room temperature, the resulting powders were collected and stored in sealed containers for further characterization.

### Characterization techniques

An X-ray diffractometer model: Siemens D500, and a field emission scanning electron microscope (FESEM) model: MIRA3-TESCAN, were used to investigate the structural characteristics and the morphology of the samples. The molecular bonds in the samples were identified using a Fourier-transform infrared (FTIR) spectrometer model JASCO-680. Raman spectra were acquired with a Horiba Jobin–Yvon Labram HR800 (excitation wavelength: 532 nm). To investigate the absorption characteristics of the samples, a PHYSTEC UVS-2500 spectrometer was used, and the optical characteristics and inherent defects of the samples were investigated using a photoluminescence (PL) spectrophotometer JASCO model FP-6200 with an excitation wavelength of 320 nm. To further probe the elemental composition, a PHI QUANTERA II XPS with an Al K-alpha source was employed. The Kol Software was utilized to fit the XPS spectra. Nitrogen adsorption–desorption isotherms were obtained using a BELSORP Mini II instrument after degassing the samples at 200 °C. The wettability of the samples was characterized by measuring the contact angle using a Jikan CAG-20 contact angle goniometer. Real-time current curves were measured to investigate the sensor characteristics using an IVIUMSTAT Potentiostat with a computer interface and an optimal applied bias of 1 V.

### Sensor fabrication and gas sensing measurement

**Fig. 1:**
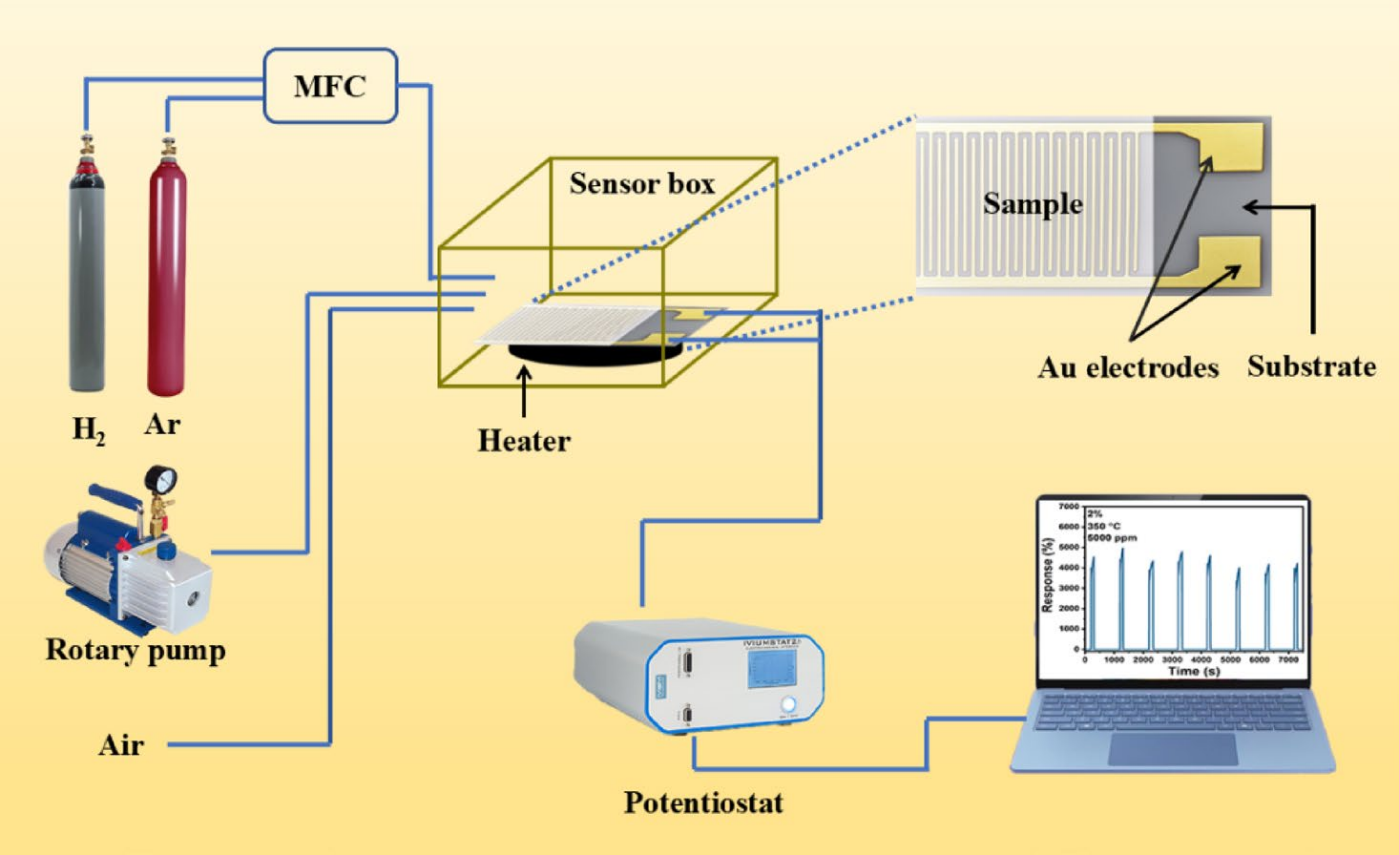
Schematic showing the gas sensor setup layout (MFC: Mass flow controller).

The general configuration for examining the gas sensor properties of the samples can be seen in Fig. 1. The nanoparticle powder was ultrasonically dispersed in DI water. The resulting solution was then deposited onto an alumina substrate coated with a gold inter-digital electrode (1 × 1 cm^2^). The electrodes were prepared by DC magnetron sputtering. The coated layer with nanoparticles (NPs) was dried overnight at room temperature. For sensing measurements in the temperature range of 150–350 °C, the samples were mounted on a compact disk-shaped PTC ceramic heating element, powered by an independent source. The temperature of the heater was calibrated for each applied voltage. The assembly was located in a chamber that was connected to the gas inlet and outlet valves. For sensing measurements, gas was injected into the chamber using an electronic shutter, and the resulting change in the sample current (I) was continuously monitored and recorded. The real-time current–time response was monitored and recorded through the Potentiostat interface connected to the computer. The gas was allowed to remain in the chamber for 100 s, and the changes in the current due to reaction with the sensor during this period were recorded. Next, the air valve was opened and the gas was removed from the chamber. As the gas was purged and air entered the chamber, the sample current returned to its initial value within a characteristic recovery time dictated by the properties of each sample. Given that the voltage applied (V) to the two ends of the sample is 1 V, the current flow resistance (R) at any moment can be calculated using the equation R = V/I. Finally, using Eq. 1, the sensor response can be obtained^30^.

1 $${\text{Response}}\,{ = }\,{\text{(R}}_{{\text{a}}} {\text{ - R}}_{{\text{g}}} {)}/{\text{R}}_{{\text{a}}} \times {100}$$

where, R_a_ indicates the resistance in air and R_g_ signifies the resistance when the target gas is present. Additionally, the sensitivity ($S$) and limit of detection (*LOD*) can be calculated using the following equations^35^:

2 $$S=\frac{\partial \left({I}_{gas}/{I}_{air}\right)}{\partial {C}_{gas}}$$

3 $$LOD=3\left(\frac{RMSD}{S}\right)$$

Here, ${C}_{gas}$ denotes the concentration of the gas, *I*_*air*_ is the base current intensity in dry air, and *I*_*gas*_ is the sensor electrical current in the presence of gas. The RMSD is the root mean square deviation of the noise signal, and the slope of the fitting line between the sensing response and the gas concentration represents the sensitivity *S* of the tested sensor.

## Results and discussion

### Structural and spectroscopic characterization

**Fig. 2:**
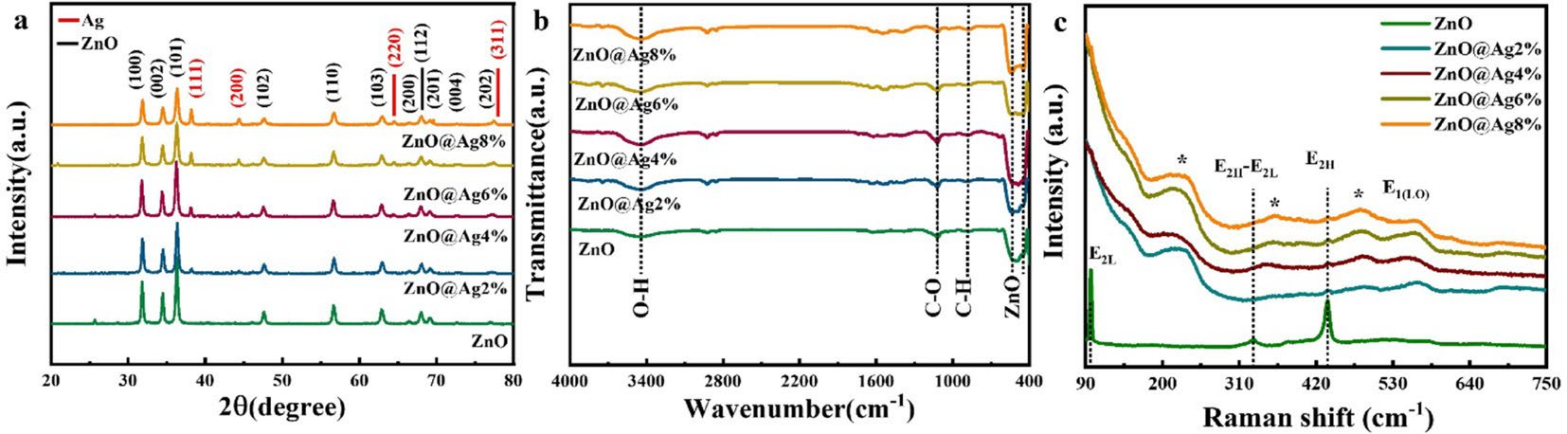
(**a**) XRD patterns, (**b**) FTIR, and (**c**) Raman spectra of ZnO@Ag samples.

First, to check the phase purity of the synthesized ZnO, the X-ray diffraction (XRD) patterns of the samples were examined. Figure 2a shows the XRD pattern of ZnO NPs with different Ag dopant percentages. The reflections corresponding to ZnO can be clearly seen in the XRD patterns. In addition, the reflections corresponding to Ag are also seen in the XRD patterns, which indicates that Ag is not completely substituted in the ZnO structure but instead occurs as a secondary phase. Apart from ZnO and Ag, no reflection corresponding to any additional phase has been detected, confirming the absence of any impurity phase in the samples. By increasing the Ag dopant percentage, the intensity of the reflections corresponding to Ag increases and this confirms the increase in the amount of Ag in the structure of the material. In addition, the intensity of the Ag (111) reflection is listed in Table S1, where it is clearly seen that the intensity of the reflection increases with increase in doping percentage. By matching the positions and relative intensities of the reflections in the experimental data with the standard pattern (no. 96–230-0113) using X’Pert HighScore software, it was confirmed that all the samples exhibit a hexagonal structure with space group P6_3_mc. Our results thus confirm the formation of ZnO phase with hexagonal structure, the presence of Ag phase, and the increasing intensity of reflections corresponding to Ag with increasing doping percentage.

The XRD data were also used to calculate the lattice constants, unit cell volumes, crystallite size, and lattice strain, and the obtained values are listed in Table S1. The lattice constants and unit cell volumes of the nanoparticles are nearly equal to the reported bulk values (a = 3.25 Å, c = 5.21 Å, and V = 47.72 Å^3^). Scherrer and Williamson-Hall (W–H) methods^36,37^ were used to calculate the crystallite size. The crystallite sizes of the pure, 2, 4, 6, and 8% Ag-doped samples were calculated to be 25, 26, 25, 23, and 21 nm using the Scherrer equation and 50, 21, 20, 18, and 17 nm using the W–H method, respectively. The W–H method is considered more accurate than the Scherrer equation, as it decouples the peak broadening caused by lattice strain and crystallite size, whereas the Scherrer method does not distinguish between strain and size^38^. Therefore, if the W–H method yields a smaller crystallite size than the Scherrer equation, it indicates that the observed peak broadening is partially due to lattice strain. Conversely, if the Scherrer size is smaller, strain effects are likely negligible. Figure S1 shows the W–H plots for the different samples. The linear fit has a positive slope for pure ZnO NPs, while the slope is negative for the doped samples. The change of sign of the slope indicates a change in the type of strain from tensile to contraction. The lattice strain values obtained from Fig. S1 are also listed in Table S1. By increasing the Ag dopant percentage, the contraction strain also increases, which can be related to the increase in the amount of Ag in the ZnO structure. The variation in strain with increasing silver doping reflects structural disorder, including local compression or stretching within the crystal lattice. Initially, up to 2% Ag doping, the strain decreases. This is attributed to the substitution of Ag^+^ ions for Zn^2+^ in the lattice. However, as the doping level increases beyond this point, the formation of a secondary silver phase is observed. At higher doping concentrations, this leads to increased structural disorder and the emergence of defects, ultimately increasing lattice strain.

To further confirm the formation of the Zn–O bond and to identify the existing bonds, FTIR spectra were recorded. Figure 2b shows the FTIR spectra of pure and Ag-doped ZnO NPs in the range of 400–4000 cm^−1^. The bands at 431 and 536 cm^−1^ arise from the Zn–O tetrahedral bond^39,40^. These peaks thus confirm the formation of Zn–O bonds. The peaks located at 882 and 1115 cm^−1^ indicate C–H and C–O bonds, respectively, which can remain in the compound through precursors or be adsorbed through the environment^37,41^. A broad peak at 3441 cm^−1^ arises from the O–H bond, which is caused by the absorption of hydroxide (–OH) groups by the samples^42–44^.

Figure 2c shows the Raman spectra of the Ag-doped ZnO NPs. The peak seen at 437 cm^−1^, which is linked to the E_2H_ mode, provides evidence for the wurtzite structure of ZnO^45,46^. The bands around 332 cm^−1^ and other typical low-frequency characteristics are likely associated with the second-order Raman spectrum originating from zone boundary phonons 3E_2H_–E_2L_^47^. The vibrational mode E_2L_ is related to the peak observed at 97.4 cm^−1^^45^. The presence of defects, such as Zn interstitial and O vacancy in the ZnO lattice, results in the E_1_(LO) modes in the range 550–560 cm^−1^. Ag incorporation in the ZnO lattice causes these defects and disorders^48^. The broad peak in the range 215–236 cm^−1^ is likely due to the radial effect of Ag atoms in the spectra. The Raman peak in the range 482–487 cm^−1^ was only observed in the Ag-doped samples because it arises from the interfacial surface phonon mode. The vibrational modes related to the Ag-doped ZnO composite may exhibit peaks in the range 349–361 cm^−1^^49^. The Raman peak shifts of the E_2H_ vibration mode to a lower wavenumber are caused by factors such as phonon confinement effect, oxygen vacancies, lattice strain, residual stress, and size effect following doping^50^.

### Morphological studies

**Fig. 3:**
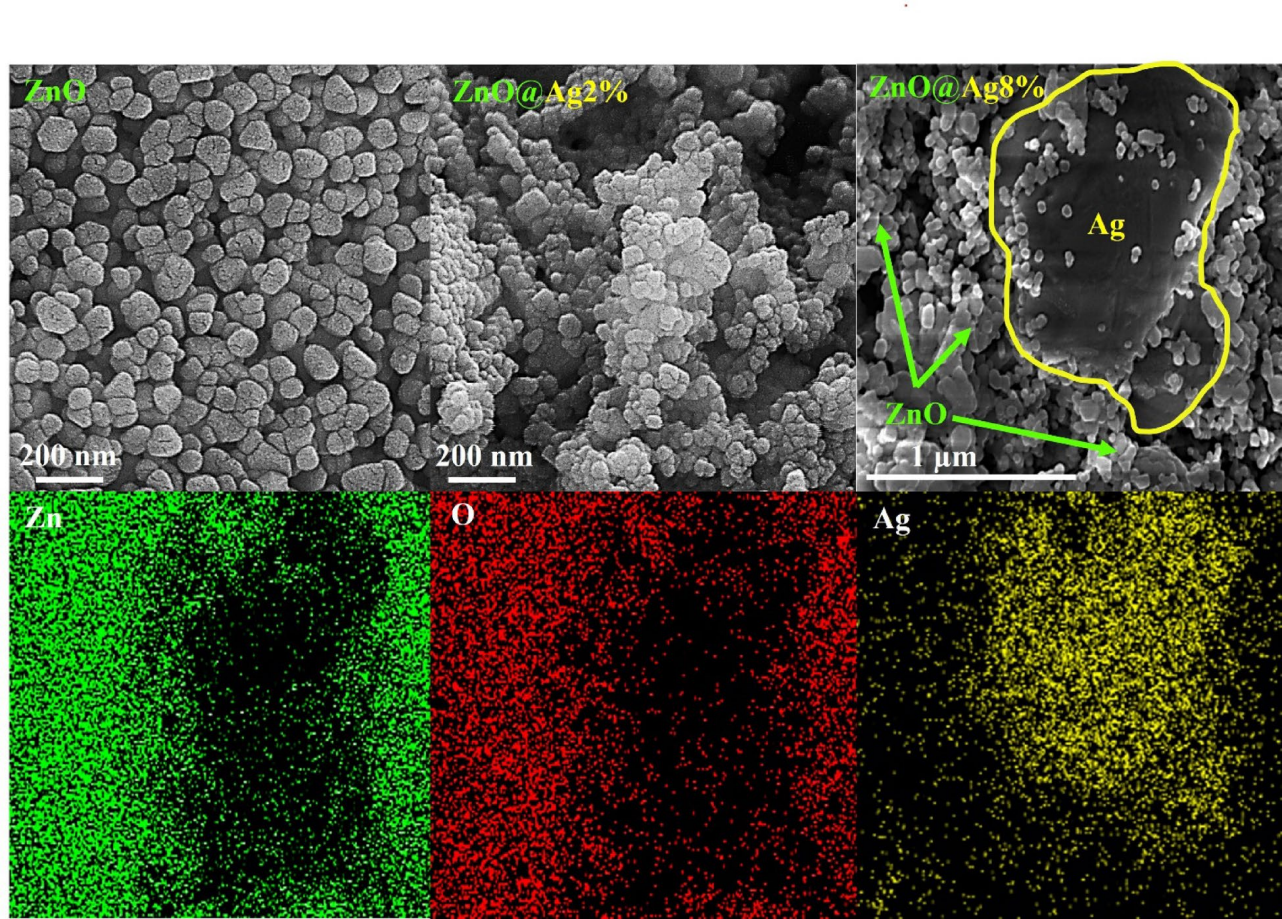
FESEM images of pure, 2%, and 8% Ag-doped ZnO NPs and elemental mapping of 8% Ag-doped ZnO NPs.

The morphology and particle size distribution of the samples were investigated using FESEM images shown in Figs. 3 and S2. The images reveal that all the samples have particles with uniform sizes. By increasing the Ag dopant concentration, the adhesion of the particles is seen to increase slightly. The elemental mapping of the samples with 8 and 2% Ag is shown in Figs. 3 and S2, respectively: it is clear that in the sample with 2% Ag, the silver is uniformly dispersed in the structure. The particle size distributions as estimated from the FESEM images are plotted in Fig. S3 and listed in Table S1. No significant change is observed in the size of the particles for different Ag doping concentrations, with the size varying in the range of 55–66 nm. Thus, the NPs are seen to have an almost uniform size distribution independent of the amount of Ag dopant, in agreement with the XRD results.

To further confirm the presence of Ag in the structure and to determine the presence of other elements, energy-dispersive X-ray (EDX) spectra were obtained from the samples (Fig. S4). The EDX spectra confirm the presence of Zn, O, Ag, and C in the samples. The atomic percentages of each element are shown as an inset for each corresponding spectrum, and the values are listed in Table S2. As expected, the atomic percent of Ag is seen to increase with an increase in the Ag dopant concentration. Thus, the EDX spectra confirm the presence of Ag, its increase with increase in dopant concentration, and agree with the results of XRD analysis.

### XPS analysis

**Fig. 4:**
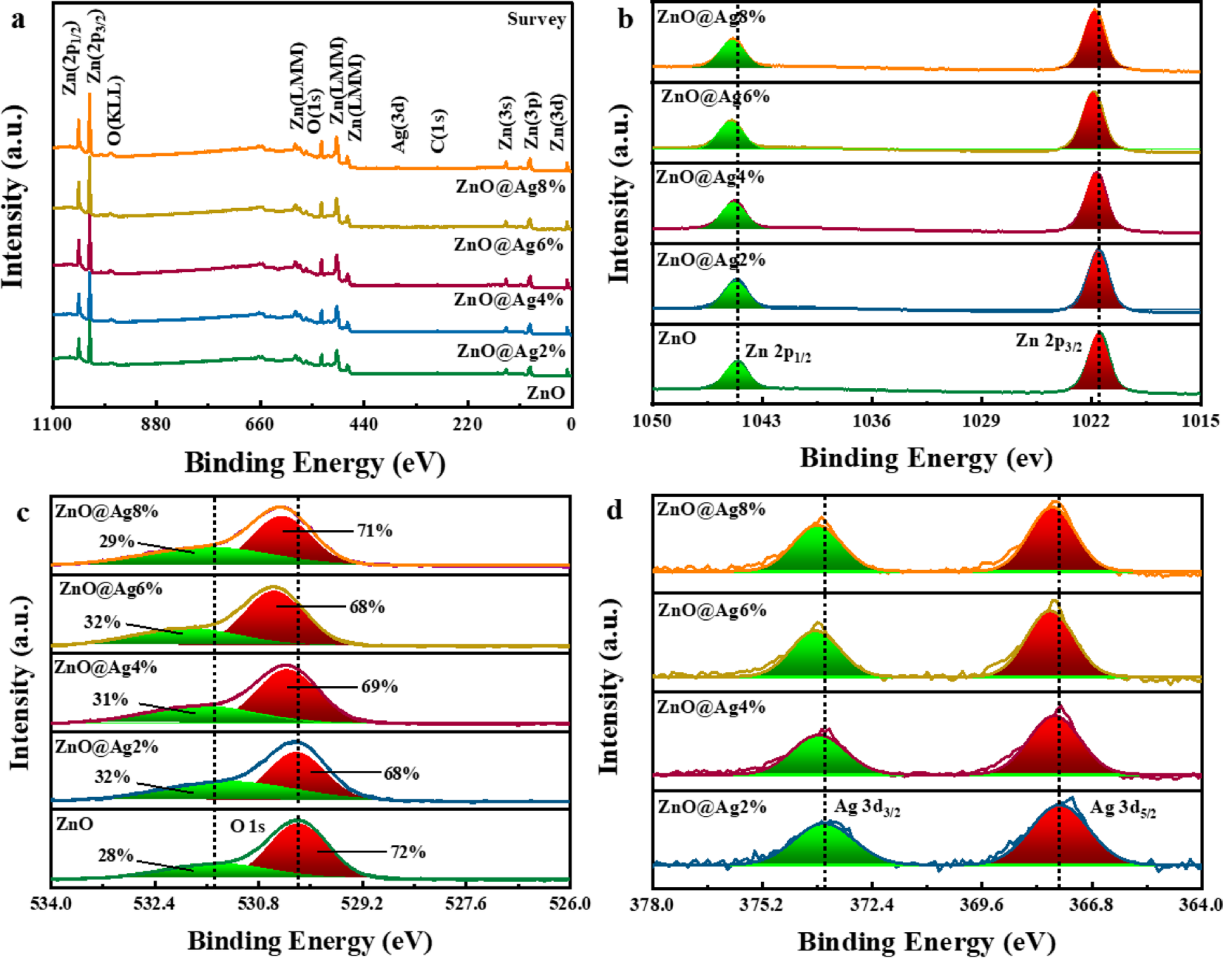
(**a**) XPS survey spectra, high-resolution XPS spectra of (**b**) Zn(2p), (**c**) O(1s), and (**d**) Ag(3d) of Ag-doped ZnO NPs.

Figures 4a–d show the XPS survey and high-resolution spectra of the NPs. The Ag-doped ZnO NPs display Zn, Ag, and O components. The carbon peak at 285 eV, labeled as C(1s), is likely caused by the adsorption of organic contaminants on the sample surface or leftover acetate. The peak values are listed in Table 1. Two peaks detected in the ranges 1021.52–1021.90 and 1044.61–1044.97 eV in the high-resolution spectra shown in Fig. 4b are identified as Zn 2p_3/2_ and Zn 2p_1/2_, respectively. They validate the Zn^2+^ oxidation level. The peaks align well with the binding energy of ZnO, indicating Zn^2+^ to O^2−^ charge transfer due to vacancies^36^. Figure 4c displays peaks corresponding to the O (1s) level in the spectra of pure and Ag-doped samples. The asymmetric O (1s) peaks can be deconvoluted into two Lorentzian-Gaussian signals, indicating the presence of two types of surface oxygen species in the samples. Both samples have lower binding energy components in the O (1s) spectra that can be attributed to O^2−^ ions in ZnO. The presence of oxygen-deficient regions is responsible for the higher binding energy components^51^. The higher binding energy peak could also be related to oxygen coming from hydroxyl groups on the surface. These hydroxyl groups on the surface can prevent the recombination of photo-generated electron–hole pairs. Ag doping causes a shift in the O (1 s) peaks towards a higher binding energy, which is a result of increased oxygen vacancies^36^. The peaks of Ag 3d_5/2_ and Ag 3d_3/2_ have binding energies of 367.61–367.86 and 373.63–373.84 eV, corresponding to metallic silver (Ag^0^) and silver ions Ag^+^, respectively, as shown in Fig. 4d^45,52^. It validates that Ag is embedded into the crystal structure of ZnO^53,54^. However, Ag is not fully embedded into the structure. The XRD data are in agreement with the XPS results.

**Table 1:** Peak positions observed in XPS spectra for different Ag doping content in ZnO NPs.

Element	ZnO	ZnO@Ag2%	ZnO@Ag4%	ZnO@Ag6%	ZnO@Ag8%
Zn 2p (eV)	1021.52	1021.58	1021.70	1021.90	1021.80
	1044.61	1044.67	1044.79	1044.97	1044.88
O 1s (eV)	530.18	530.22	530.36	530.56	530.44
	531.56	531.55	531.77	531.97	531.79
Ag 3d (eV)	–	367.61	367.75	367.86	367.81
		373.63	373.75	373.84	373.78

### Optical properties

**Fig. 5:**
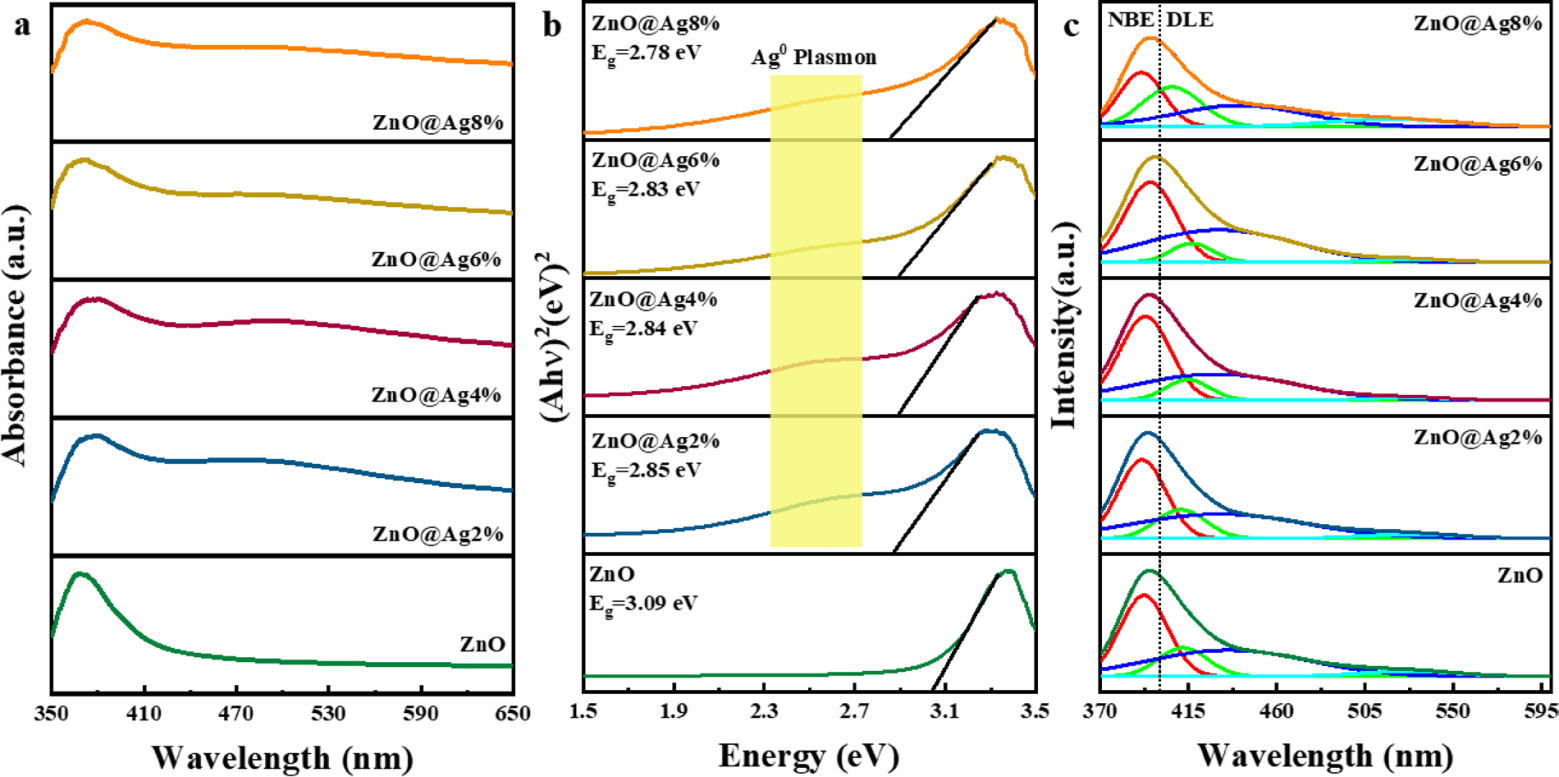
(**a**) UV–Vis absorption, (**b**) Tauc plots, and PL spectra of ZnO NPs with different Ag doping levels.

To study the absorption characteristics and calculate the band gap energy, the UV–Vis spectra of the samples were examined. In Fig. 5a, we show the absorption spectra of all the samples. Pure ZnO NPs do not absorb in the visible region and are transparent; in the ultraviolet region, the absorption is seen to increase, and the NPs act as ultraviolet light absorbers. The Ag-doped ZnO NPs also absorb in the ultraviolet region. Figure 5b shows Tauc’s plots that were used to calculate the band gap energy. In the figure, a broad peak can be seen in the samples doped with Ag, which is caused by the silver surface plasmon polaritons^55,56^. The band gap energy values calculated using Tauc’s equation are listed in Table 2. It is seen that on increasing the concentration of Ag dopant, the band gap energy decreases. The reduction of the band gap energy is related to the change in the band structure. Ag, as a shallow electron-accepting impurity, creates an energy state near the edge of the valence band^57^. By increasing the amount of Ag dopant, the density of the energy states created increases, and this reduces the band gap. Moreover, the interaction between the energy states of Ag and ZnO can create new energy states in the band gap region, which ultimately leads to the reduction of the band gap energy^58^. Thus, the samples have maximum absorption in the ultraviolet region, and by increasing the content of dopant, the band gap decreases, which is due to the formation of energy states near the valence band.

**Table 2:** Band gap energy obtained from the UV–Visible spectra and the location of peaks related to the NBE and to DLE (Zn_i_, V_O_) emission obtained from the PL spectra.

Parameter	ZnO	ZnO@Ag2%	ZnO@Ag4%	ZnO@Ag6%	ZnO@Ag8%
Bandgap (eV)	3.09	2.85	2.84	2.83	2.78
NBE (nm)	390.8	392.7	393.4	395.9	395.3
Zn_i_ (nm)	406.8	412.8	414.2	417.1	416.5
V_O_- Zn_i_ (nm)	440.9	433.3	430.8	435.3	430.7
V_O_ (nm)	517.6	526.6	528.3	527.9	529.4

The optical properties and inherent defects of the samples were analyzed using Photoluminescence (PL) spectra. Figure 5c shows the PL spectra of NPs with 0, 2, 4, 6, and 8% Ag dopant along with their Gaussian fits. As seen in the figure, the peaks related to near band edge emission (NBE) are higher in intensity than the peaks related to deep levels emission (DLE), and this shows that the samples have better crystal quality^30,59^. The peak positions obtained from the Gaussian fit for the samples are listed in Table 2. The peaks located in the range of less than 400 nm are related to NBE emission^37^. As the amount of Ag dopant increases, the NBE peak of the samples shifts to longer wavelengths (lower energies). The broad peaks in the range of 400 to 600 nm are related to DLE emission, which is caused by inherent defects in the NPs. The peaks located at 406.8, 412.8, 414.2, 417.1, and 416.5 nm are due to interstitial zinc atoms^37,60^. The blue emission in the range of 430 to 440 nm is related to oxygen atom vacancies or interstitial zinc atoms^17^. The green emission in the range of 517 to 530 nm is caused by oxygen atom vacancies^61^. In summary, the intensity of NBE peaks was higher than that of DLE, and this confirms the superior crystalline quality of the samples. Moreover, the peak corresponding to NBE of the samples shifts to lower energies with an increase in Ag dopant concentration.

### BET and wettability analyses

**Fig. 6:**
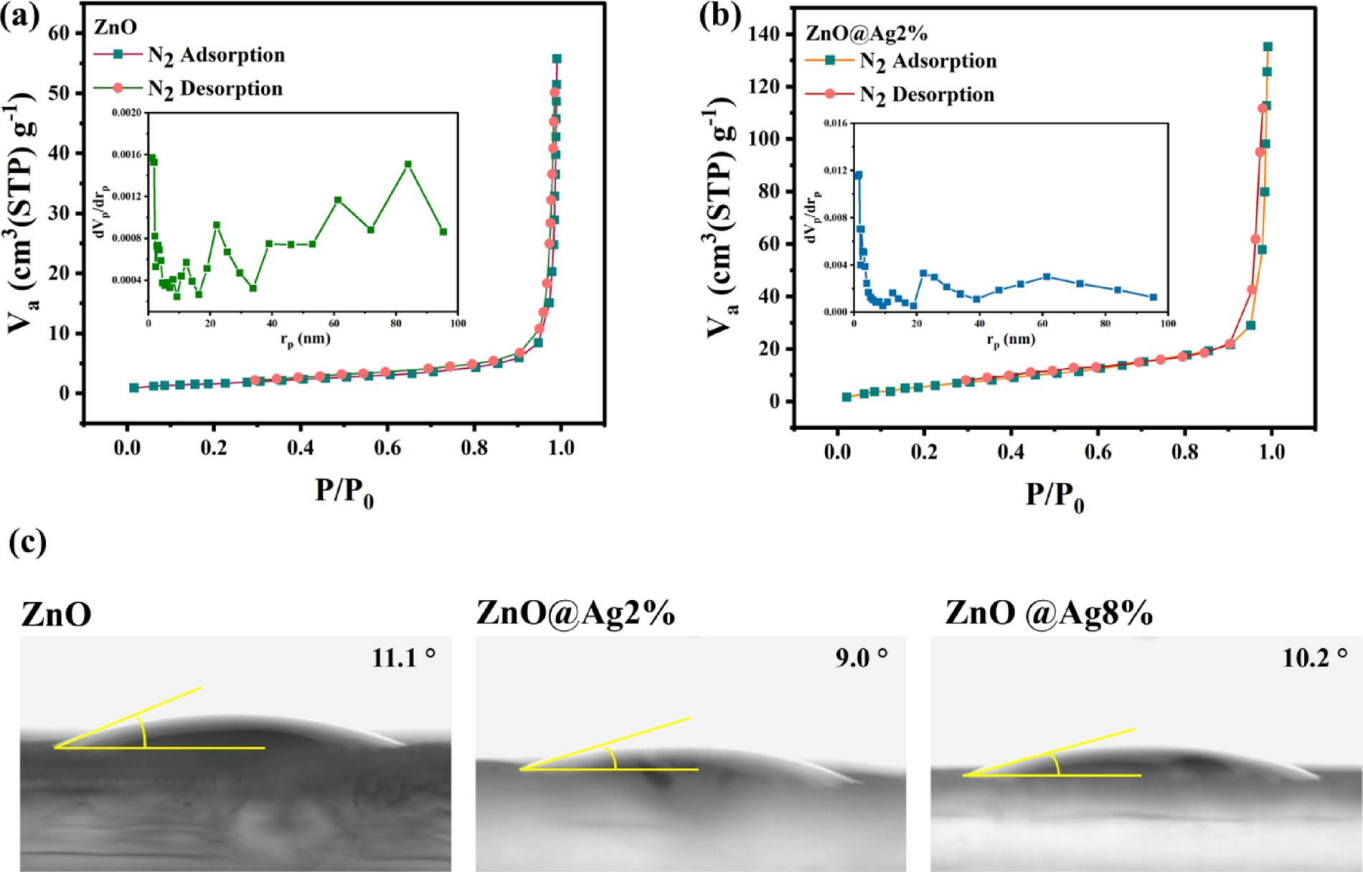
N_2_ adsorption–desorption isotherm and BJH pore size distributions (inset) of (**a**) ZnO, (**b**) 2% Ag-doped ZnO NPs, and (**c**) wettability of ZnO, 2%, and 8% Ag-doped NPs.

The surface structure, pore type, diameter, and distribution of NPs play a crucial role in the sensing performance of materials. To evaluate the porosity of the samples, BET analysis was conducted on pure ZnO and 2% Ag-doped ZnO NPs. Figure 6a, b present the nitrogen adsorption–desorption isotherms and the corresponding BJH pore size distribution plots. According to the IUPAC classification, the observed isotherms are characteristic of mesoporous materials^37^. The specific surface areas of pure ZnO and 2% Ag-doped ZnO were found to be 6.23 and 26.48 m^2^/g, respectively. The BJH analysis (inset) shows that ZnO possesses pores with radii ranging from 20 to 100 nm, while the Ag-doped sample exhibits narrower distributions centered around 25 and 60 nm. Furthermore, the vertical axis of the BJH plots indicates that the number of pores in the Ag-doped ZnO is significantly higher than in pure ZnO. This increase in pore quantity and surface area provides more active sites for gas adsorption, thereby enhancing the sensing response and improving both the response and recovery times.

Figure 6c presents the static contact angles of pure ZnO and Ag-doped ZnO NPs with 2% and 8% Ag content. ZnO is inherently hydrophilic^62^ and all three samples exhibit hydrophilic behavior. Among them, the pure ZnO sample shows the highest contact angle, indicating relatively higher surface tension and lower surface energy^4^. Upon doping with 2% Ag, the contact angle decreases, which may be attributed to a reduction in crystallinity induced by the dopant^63^. Interestingly, when the Ag doping increases to 8%, the contact angle increases again. The observed increase in contact angle at 8% doping can be attributed to the formation of Ag as a secondary phase, as metallic silver is known to exhibit hydrophobic behavior.

### Gas-sensing properties

**Fig. 7:**
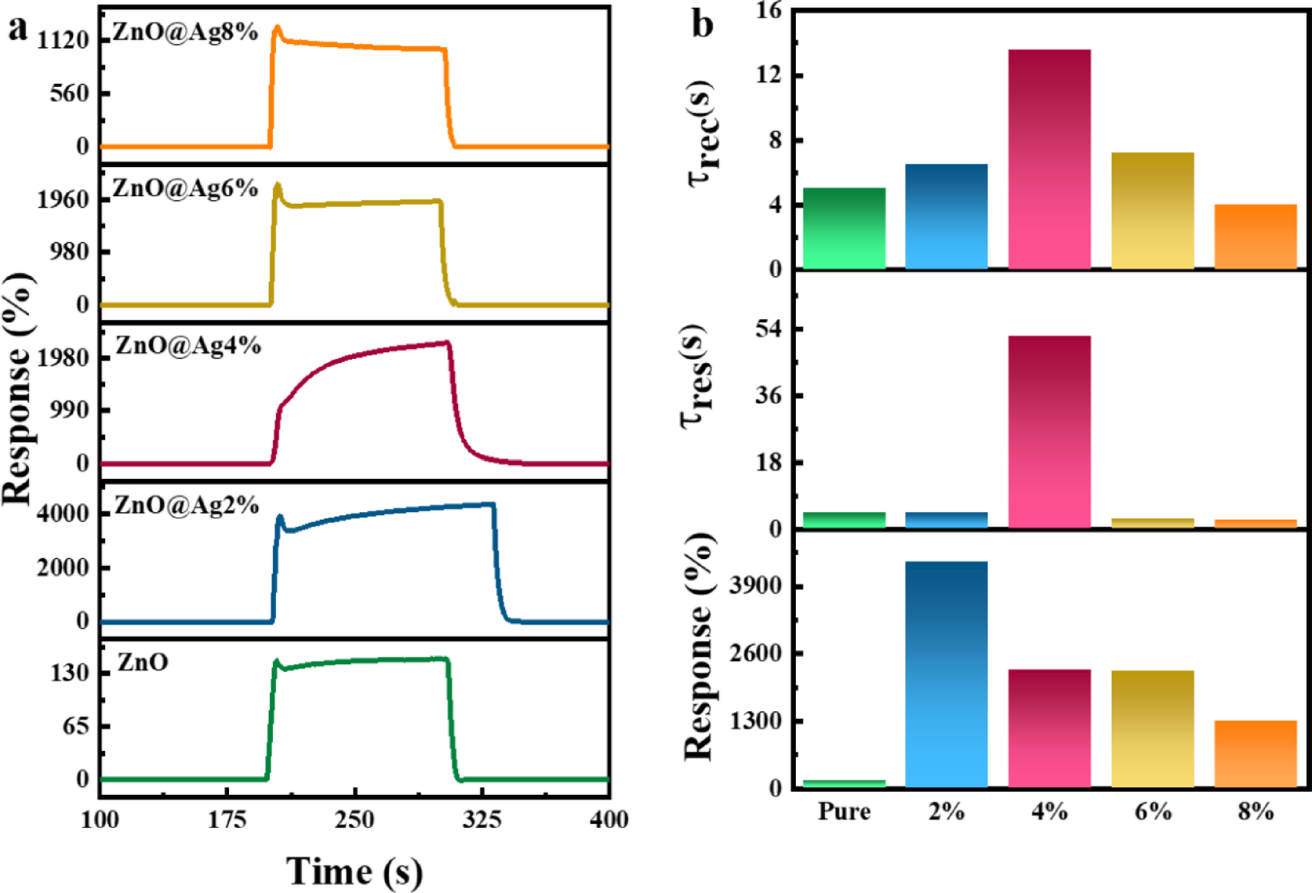
(**a**) Response plots versus time, (**b**) response time, recovery time, and response values of the Ag-doped ZnO sensors. The data were recorded at 350 °C and 5000 ppm H_2_.

Figure 7a shows the variation of percentage response as a function of time recorded at 350 °C and a hydrogen gas concentration of 5000 ppm for the ZnO NPs with 0, 2, 4, 6, and 8% Ag dopant.

The response and recovery times and % response values are shown in Fig. 7b and listed in Table 3. On increasing the silver content to 2%, the % response improved due to the spill-over effect. Spill-over refers to the availability of more electrons, which leads to proper resistance control. However, with increasing amount of silver in the sample, the spill-over effect decreased due to the agglomeration of NPs^64^. The agglomeration of NPs on increased Ag doping was confirmed previously from the FESEM images. On the other hand, the FESEM images also showed that the 2% Ag-doped sample has a smaller particle size than the other samples (see Table S1). This leads to an increase in the surface-to-volume ratio, which in turn increases the rate of nanoparticle reaction with the target gas and improves the sensor properties. As seen in Fig. 7(a), the samples with 2 and 4% Ag doping have a higher % response compared to the other samples.

**Table 3:** Response (${\tau }_{res}$) and recovery (${\tau }_{rec}$) times of the samples exposed to H_2_ gas.

Parameter	ZnO	ZnO@Ag2%	ZnO@Ag4%	ZnO@Ag6%	ZnO@Ag8%
Response (%)	149	4357	2272	2252	1280
${\tau }_{res}$(s)	4.3	4.3	52	2.7	2.5
${\tau }_{rec}$(s)	5.0	6.5	13.6	7.2	4.0

According to previous studies, the presence of a silver phase within the ZnO structure can enhance the response and recovery times by increasing both the H_2_ gas adsorption capacity and the diffusion rate^30^. In the present study, a reduction in response and recovery times is observed in samples doped with 6 and 8% Ag, which is attributed to improved H_2_ adsorption and diffusion facilitated by the catalytic effect of the silver phase. However, at lower doping levels (0–4%), the response and recovery times increase with increasing Ag content. Specifically, at 2% Ag doping, XRD analysis indicates that most Ag^+^ ions substitute for Zn^2+^ in the crystal lattice, which does not significantly disturb the lattice order. As the doping level increases to 4%, the excess Ag^+^ ions that do not enter the lattice form a secondary phase with irregular morphology. This secondary phase introduces structural defects and electron traps, which hinder charge transport and delay surface reactions, resulting in increased response and recovery times. The 4% Ag doping level represents a critical threshold. At this concentration, the silver phase has not yet developed sufficient catalytic properties. As a result, unlike higher doping levels (6 and 8%), it cannot effectively enhance H_2_ gas adsorption or diffusion processes.

Thus, the sample with 8% Ag doping has the lowest response and recovery times; however, it also has a lower % response compared to the samples with 2, 4, and 6% Ag doping. Therefore, the 2 and 4% Ag-doped ZnO NPs with their superior % response and relatively good response and recovery times were chosen for further studies.

**Fig. 8:**
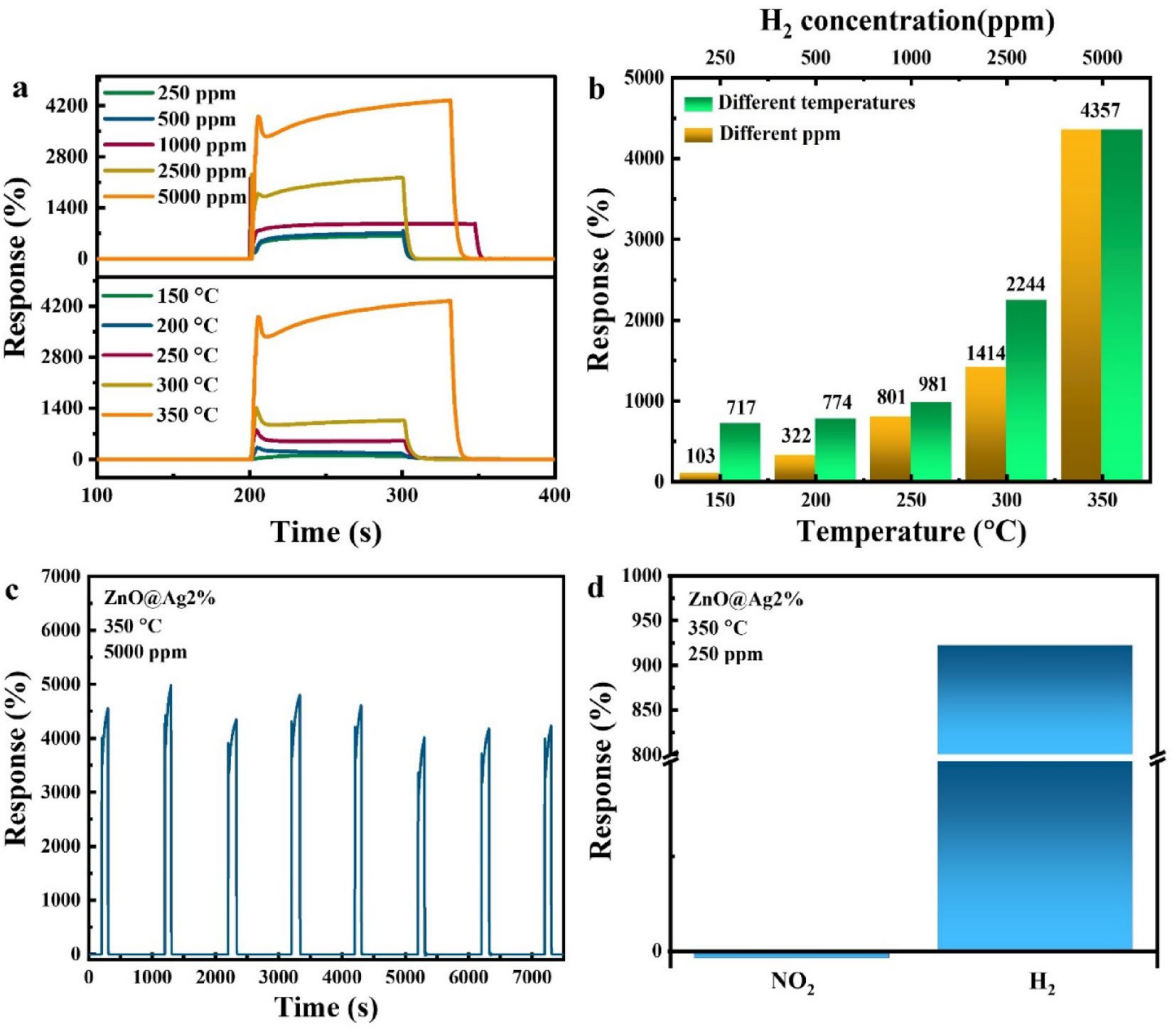
Percentage response at different (**a**, **b**) temperatures and gas concentrations, (**c**) iterations, and (**d**) selectivity of the 2% Ag-doped ZnO sensor.

After selecting the 2% Ag-doped sample as the best gas sensor, its sensing response at different temperatures and concentrations of H_2_, stability, and selectivity were investigated first. Figure 8a shows the variation of % response as a function of time for the sample with 2% Ag doping for different temperatures and concentrations of hydrogen gas. The % response values are also shown in Fig. 8b. As can be seen from Fig. 8a, the % response increases with an increase in temperature and concentration of H_2_. To investigate the stability, repeatability tests were performed on the sample. This is shown in Fig. 8c for the 2% Ag-doped sample at 350 °C with a concentration of 5000 ppm. As can be seen from the figure, even after 8 consecutive exposure cycles, the sample maintains its % response and sensing characteristics. As seen in Fig. 8c, the response exhibits some fluctuations; however, it always exhibits a value greater than 4000%, which indicates that the ZnO nanostructures are capable of storing H_2_ gas. The slight fluctuations can be understood from the sequential introduction of H_2_ and air in the chamber. When H_2_ is introduced into the chamber, the sensor adsorbs and partially stores H_2_ gas. In the subsequent step, when air is introduced, the adsorbed H_2_ desorbs gradually. However, before the desorption process is fully completed, H_2_ gas is reintroduced into the chamber, leading to a decrease in the sensor response due to the presence of residual H₂ from the previous cycle^65,66^. The % response of the sample in the presence of two different gases, H_2_ and nitrogen dioxide (NO_2_), was also investigated. This is shown in Fig. 8d for the 2% Ag-doped sample at 350 °C and 250 ppm concentration. The maximum % response values are observed to be 922 and − 2 for H_2_ and NO_2_ gas, respectively. Notably, the % response rate is positive for H_2_ gas and negative for NO_2_ gas. The negative response for NO_2_ can be attributed to the fact that it is an oxidizing gas^28^.

H_2_ gas is more effectively adsorbed on the ZnO surface compared to other gases. This is primarily due to the strong interaction between H_2_ molecules and chemisorbed oxygen ions on the ZnO surface, leading to the formation of O–H bonds. This reaction releases electrons back into the conduction band of ZnO, thereby increasing the carrier concentration. As a result, a metallic surface layer forms, reducing the sensor’s resistance and significantly enhancing its response to H_2_. In contrast, for NO_2_ gas, the metallization process does not occur. Instead, NO_2_ acts as a strong electron acceptor, further depleting the conduction electrons in ZnO. This leads to an increase in the sensor resistance and results in a comparatively lower response for NO_2_^67^.

**Fig. 9:**
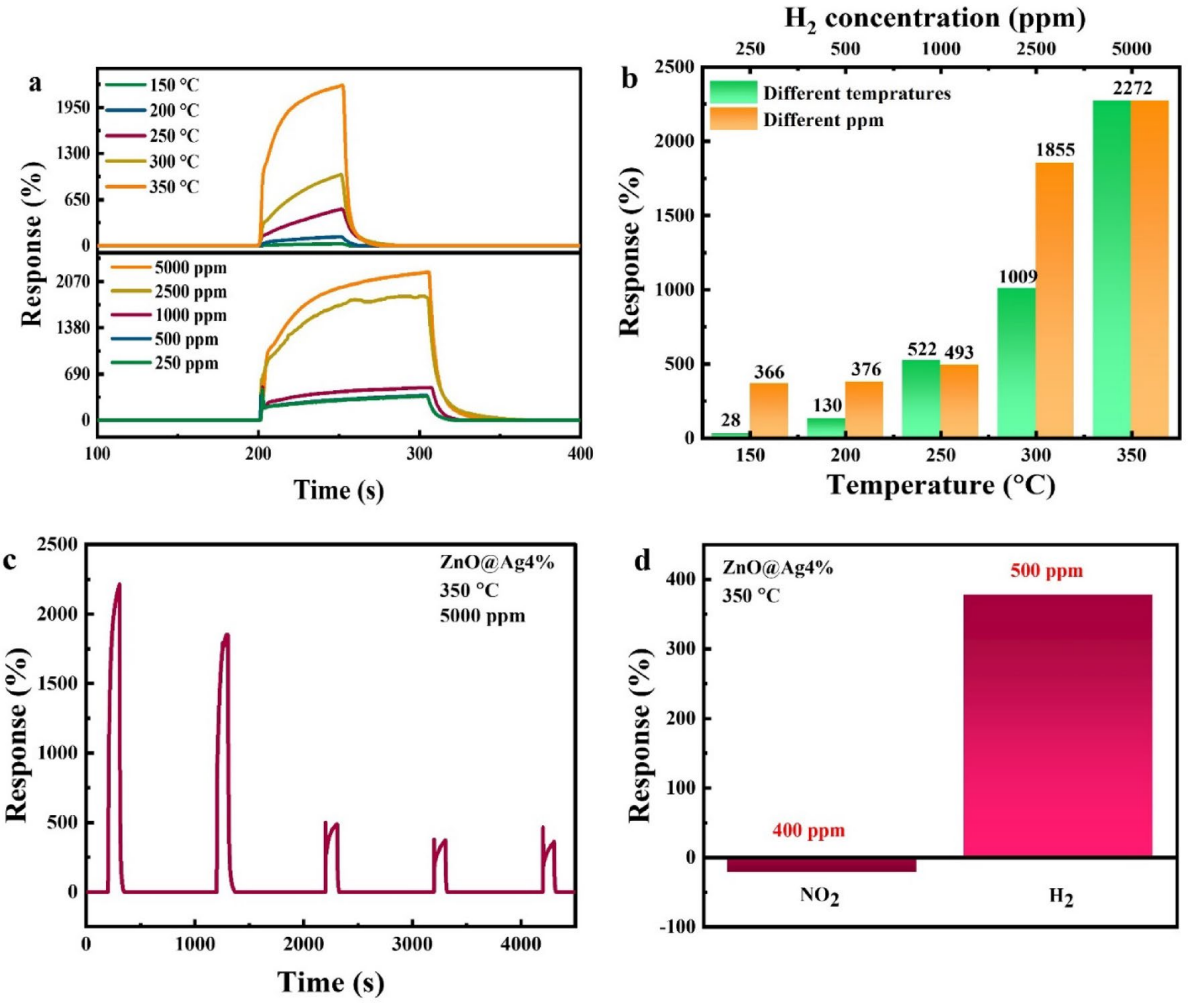
Percentage response at different (**a**,**b**) temperatures and gas concentrations, (**c**) iterations, and (**d**) selectivity of the sensor based on 4% Ag-doped ZnO NPs.

Next, the % response of the sample with 4% Ag doping at different temperatures and concentrations, as well as its stability and selectivity, were investigated. Figure 9a shows the variation of % response as a function of time for different temperatures and gas concentrations. It is clear from the figure that the % response increases with both increasing temperature and gas concentration. The % response values at different temperatures and gas concentrations are also shown in Fig. 9b, where it can be seen that the sample exhibits the highest response at 350 °C and for 5000 ppm gas concentration. The stability test for this sample (Fig. 9c) reveals that the % response decreases significantly from the first to the third iterations. However, for subsequent iterations, the % response becomes stable. For the selectivity test, the % response of the sample was investigated in the presence of two different gases, H_2_ and NO_2_, at a temperature of 350 °C and gas concentrations of 500 and 400 ppm, respectively (Fig. 9d). Percentage response values of 378 and − 22 were observed for H_2_ and NO_2_, respectively. Thus, similar to the 2% Ag-doped sample, the 4% Ag-doped ZnO NPs also exhibit a positive response for H_2_ gas and a negative response for NO_2_ gas.

Moreover, the *S* and *LOD* values were calculated using Eqs. 2 and 3 for the 2 and 4% Ag-doped ZnO NPs. The sensitivities (*S*) were found to be 0.787 and 0.443 ppm⁻^1^ for the 2 and 4% Ag-doped samples, respectively, indicating that the 2% Ag-doped sensor exhibits higher sensitivity compared to the 4% Ag-doped sensor. The *LOD* values were determined to be 0.49 ppb and 0.68 ppb for the 2% and 4% Ag-doped ZnO NPs, respectively. These results demonstrate that the sensors are capable of detecting very low concentrations of target gas and exhibit high sensitivity to concentration changes^68^.

### Gas sensing mechanism

**Fig. 10:**
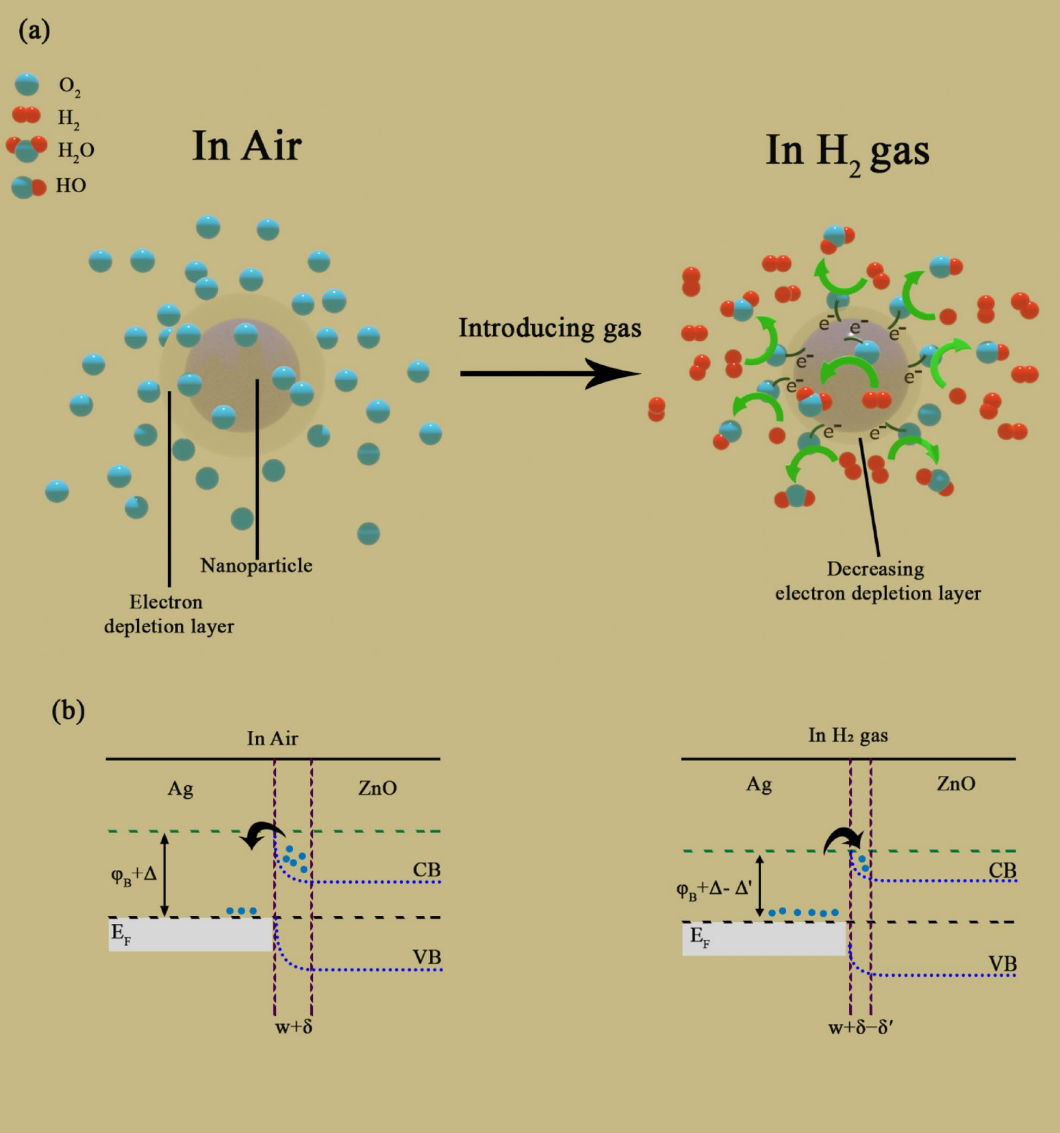
Schematic illustration of (**a**) gas sensing mechanism and (**b**) band structure of Ag-doped ZnO sensor.

The sensing mechanism of NP-based gas sensors depends on the physical and chemical processes that occur on the surfaces of the NPs. A schematic illustrating this process is shown in Fig. 10a. When a chemo-resistive type sensing material interacts with a target gas, it results in a noticeable variation in the material’s resistance. ZnO is an n-type semiconductor with the majority of charge carriers being electrons. When air is present, oxygen in the environment reacts with the ZnO surface, moves through the grain boundaries, and is absorbed at both the surface and grain boundaries. The absorbed oxygen accepts electrons from the conduction band of ZnO, forming ionic oxygen species (O_2_^−^, O^−^, and O^2–^) depending on the temperature. At different temperatures, the oxygen molecules adsorbed on the sensor surface are different (see Eqs. 1 to 4 in the supplementary information).

The adsorbed oxygen species trap electrons in the metal oxide and form an electron depletion region near the surface, causing band bending, which increases the resistance of the sensor^69^. In this work, the optimal operating temperature was found to be 350 °C, which means that the majority of the oxygen molecules are ionized to ${\text{O}}^{-}$ and ${\text{O}}^{{2}-}$. According to Eqs. 5 to 7 in the supplementary information, with the formation of water molecules and hydroxyl radicals in the presence of hydrogen gas, the trapped electrons are released and the resistance of the sensor is reduced. The decrease in resistance is due to the decrease in the height of the potential barrier, the decrease in the width of the depletion layer, and the return of electrons to the conduction band. The decrease in resistance can also be caused by surface metallization due to the reaction of hydrogen gas with ZnO and the conversion of ZnO to Zn at the surface of the sensor^30^. The selectivity for H_2_ gas increases in Ag-doped ZnO NPs. This could be linked to the chemical and electronic sensitization when Ag NPs are present. Ag NPs in chemical sensitization led to a spill-over effect, boosting the presence of adsorbed oxygen species and enhancing the catalytic effect on the reaction between H_2_ gas and these species. Moreover, the spill-over effect improves the rate of hydrogen diffusion on ZnO by converting hydrogen gas into hydrogen atoms, thereby increasing the % response.

The modulation of the charge depletion layer can be used to describe electronic sensitization. In the presence of Ag NPs on the ZnO surface, the depletion layer width near the metal–semiconductor interface increases. The energy required for the work function of Ag is approximately 4.26 eV^70^, while for ZnO it is 4.45 eV^71^. Due to this discrepancy in work function, electrons are directed towards the silver from the conduction band of ZnO, forming a Schottky barrier at the Ag–ZnO interface, leading to an increase in resistance when oxygen is present. In the presence of H_2_ gas, the height of the Schottky barrier decreases, thereby decreasing the depletion layer width and releasing electrons to the conduction band of ZnO. This reduction enhances the response, particularly in Ag-doped ZnO samples compared to pure ZnO^30^.

Figure 10b illustrates the formation of a Schottky contact between ZnO and Ag. In air, electrons are transferred from ZnO to the Ag surface due to the difference in work functions. Additionally, some electrons from ZnO are captured by oxygen molecules adsorbed on the surface, forming negatively charged oxygen species (e.g., O⁻ or O_2_⁻). This process leads to the formation of an electron-depleted region near the ZnO surface, commonly referred to as the depletion layer (w + δ). It is accompanied by an upward band bending. Additionally, the potential barrier height increases (φ_B_ + Δ).

Upon exposure to H_2_ gas, silver acts as a catalyst and facilitates the dissociation and activation of oxygen species via the spill-over effect. These active oxygen species migrate to the ZnO surface, where H_2_ molecules react with the adsorbed oxygen ions, forming water and releasing the previously trapped electrons back into ZnO. As a result, the depletion layer shrinks (w + δ − δ′), the potential barrier is reduced (φ_B_ + Δ − Δ′), and the conductivity of ZnO increases. This change enhances the overall sensing performance of the Ag–ZnO structure toward H_2_ gas^30^.

## Conclusion

In this work, Ag-doped ZnO NPs were prepared by thermal decomposition, and their properties were investigated for use in the field of gas sensing. The chemical and physical properties of the samples were investigated using XRD, XPS, FT-IR, and Raman experiments. The presence of a hexagonal crystal structure, Zn–O bonds, and silver in the samples was confirmed. Through UV–Vis and PL measurements, a reduction in the band gap energy and the presence of defects in the structure were observed. Gas sensor experiments were conducted for the samples to detect hydrogen gas, and the samples with doping amounts of 2 and 4% Ag were observed to exhibit the highest % response. These two samples were also tested at higher temperatures and gas concentrations, and it was found that the % response increases as the temperature and gas concentration increase. In addition to hydrogen gas, NO_2_ gas was also tested for the samples with 2 and 4% Ag doping. This study introduces a significant advancement in the development of Ag-doped ZnO NPs, demonstrating that the sample with 2% Ag doping shows the highest potential for gas sensing applications. These results not only validate the effectiveness of controlled doping but also open new avenues for future research in the simple design and rapid engineering of high-performance gas sensors.

## Supplementary Information

Below is the link to the electronic supplementary material.

Supplementary Material 1

## Data Availability

The datasets used and/or analysed during the current study available from corresponding author on reasonable request.
