# Supplementary material for: High-performance hydrogen gas sensor based on Ag-incorporated ZnO nanoparticles
Source: Sci Rep. 2025 Nov 3;15:38390. doi: 10.1038/s41598-025-22222-9 (PMC12583680; doi:10.1038/s41598-025-22222-9)
Supplement: Supplementary file 1 — Supplementary Material 1 [file 41598_2025_22222_MOESM1_ESM.docx]

**High performance H_2_ gas sensor based on Ag doped ZnO nanoparticles**

**Reza Torkamani^1^, Bagher Aslibeiki^1,2^**^*^**, Saeid Salari^3,4^, Hamid Azizi^1^, Davide Peddis^5,6^ & Tapati Sarkar^2^**^**^

^1^Faculty of Physics, University of Tabriz, Tabriz, Iran

^2^Department of Materials Science and Engineering, Uppsala University, Box 35, Uppsala SE-75103, Sweden

^3^Department of Physics, Isfahan University of Technology, Isfahan, 84156-83111, Iran

^4^RCQI, Institute of Physics, Slovak Academy of Sciences, Dúbravská cesta 9, 84511 Bratislava, Slovakia

^5^Department of Chemistry and Industrial Chemistry & Genova, INSTM RU, nM2-Lab, University of Genova, 16146 Genova, Italy

^6^Institute of Structure of Matter, National Research Council, nM2-Lab, Via Salaria km 29.300, Monterotondo Scalo 00015, Roma, Italy

**E-mail:* [*b.aslibeiki@tabrizu.ac.ir*](mailto:b.aslibeiki@tabrizu.ac.ir)

***E-mail:* [*tapati.sarkar@angstrom.uu.se*](mailto:tapati.sarkar@angstrom.uu.se)

**Gas sensor mechanism in Ag-doped ZnO NPs**

When the sensor is exposed to air, oxygen molecules are adsorbed onto the sensor surface at different temperatures: $\text{O}_{\text{2}}^{\text{-}}\text{ }$(<150 °C), $\text{O}^{\text{-}}\text{ and }\text{O}^{\text{2-}}$(>150 °C) as shown in the following equations^1,2^:

$\text{O}_{\text{2}}\text{→}\text{O}_{\text{2 (ads)}}$ (1)

$\text{O}_{\text{2 (ads)}}\text{+ e}^{\text{-}}\text{→}\text{O}_{\text{2 (ads)}}^{\text{-}}$ (2)

$\text{O}_{\text{2 (ads)}}^{\text{-}}\text{+ e}^{\text{-}}\text{→}\text{2O}_{\text{ (ads)}}^{\text{-}}$ (3)

$\text{O}_{\text{ (ads)}}^{\text{-}}\text{+ e}^{\text{-}}\text{→}\text{O}_{\text{ (ads)}}^{\text{2-}}$ (4)

When H_2_ gas is introduced, H molecules are oxidized by $\text{O}^{\text{-}}$ and $\text{O}^{\text{2-}}$ to form H_2_O and HO, as shown in equations 6 and 7^3^.

$\text{H}_{\text{2 (g)}}\text{+}\text{e}^{\text{-}}\text{→}\text{H}_{\text{2 (ads)}}\text{→}\text{2H}_{\text{ (ads)}}^{\text{+}}$ (5)

$\text{H}_{\text{ (ads)}}^{\text{+}}\text{+}\text{O}_{\text{ (ads)}}^{\text{-}}\text{→}\text{HO}_{\text{ (vap)}}$ (6)

$\text{2H}_{\text{ (ads)}}^{\text{+}}\text{+}\text{O}_{\text{ (ads)}}^{\text{2-}}\text{→}{\text{H}_{\text{2}}\text{O}}_{\text{ (vap)}}$ (7)


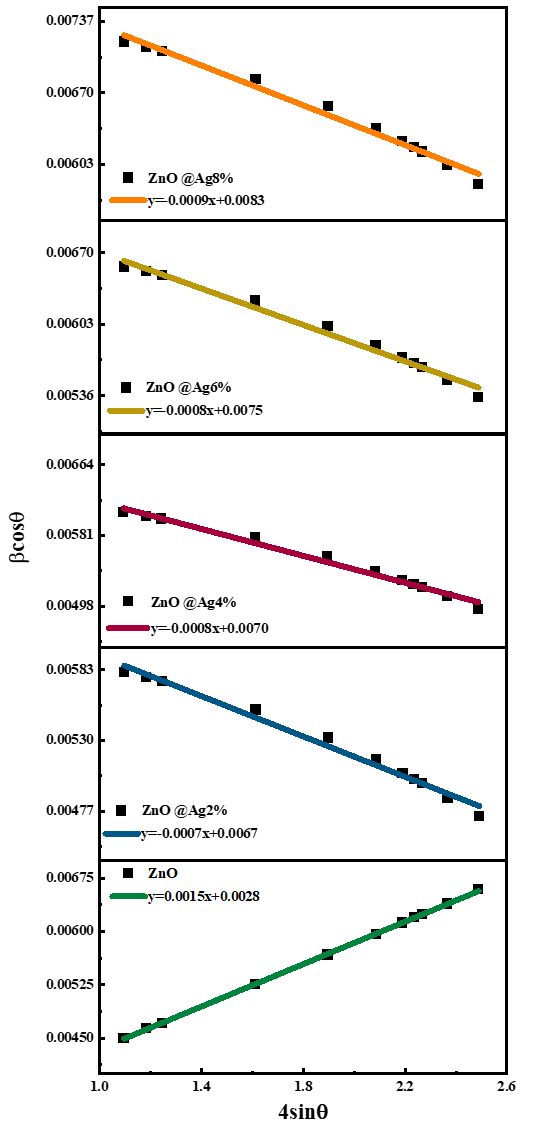


**Fig. S1.** W-H plots of ZnO NPs with different Ag doping percentage.


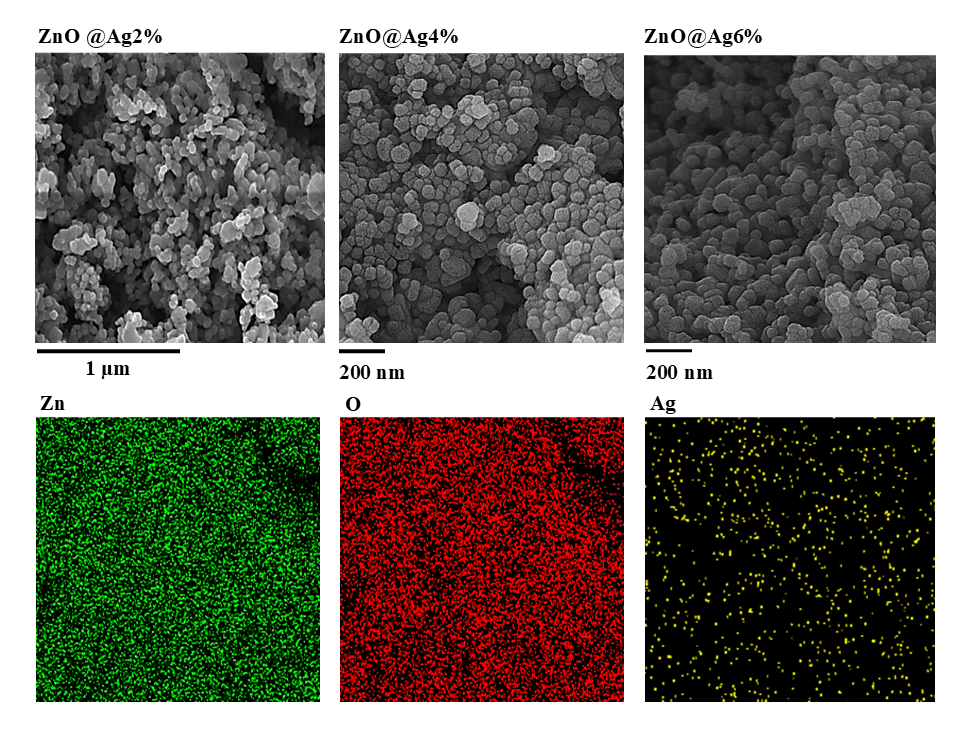


**Fig. S2.** FESEM images of NPs and elemental mapping for 2% Ag-doped ZnO NPs.


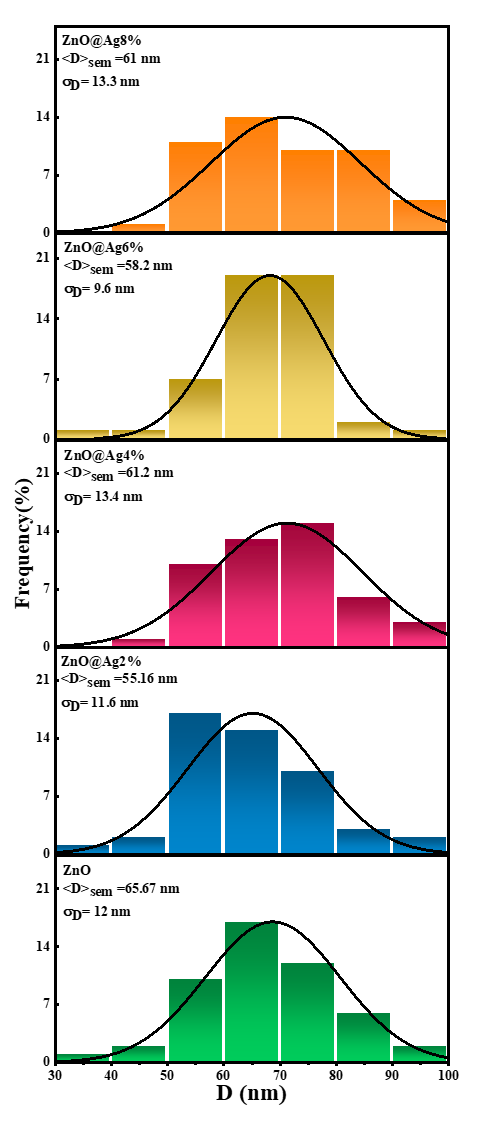


**Fig. S3.** Particle size distribution of ZnO NPs with different Ag doping concentrations.


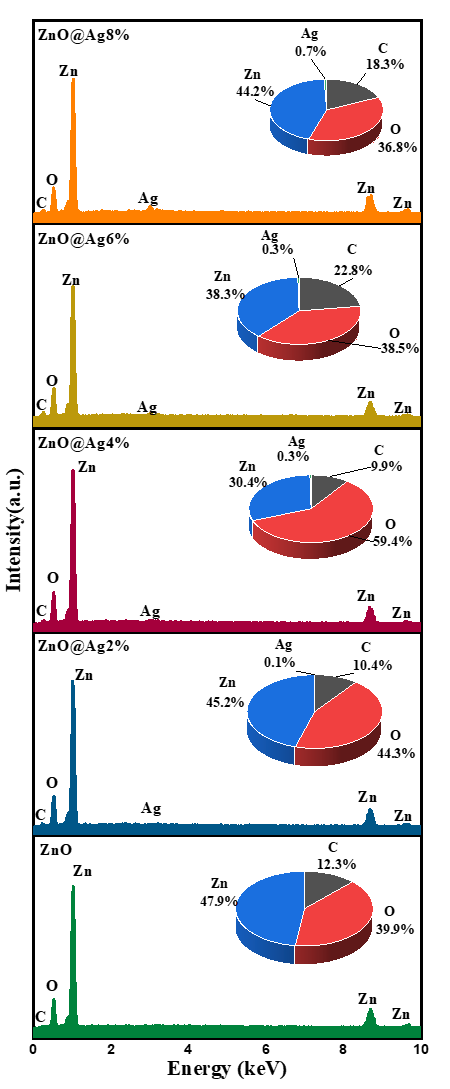


**Fig. S4.** EDX spectra of Ag-doped ZnO NPs.

**Table S1.** Intensity of Ag (111) peak, lattice constants, unit cell volume, crystallite size, strain, and particle size of Ag doped ZnO NPs.

| **Parameter** | **ZnO** | **ZnO@Ag2%** | **ZnO@Ag4%** | **ZnO@Ag6%** | **ZnO@Ag8%** | |
| --- | --- | --- | --- | --- | --- | --- |
| Intensity  Ag (111) peak | - | 248 ± 0.2 | 446 ± 0.2 | 585 ± 0.3 | | 707 ± 0.3 |
| a (Å) | 3.25 ± 0.0010 | 3.24 ± 0.0010 | 3.25 ± 0.0003 | 3.25 ± 0.0013 | | 3.24 ± 0.0019 |
| c (Å) | 5.20 ± 0.0020 | 5.20 ± 0.0020 | 5.21 ± 0.0006 | 5.20 ± 0.0023 | | 5.20 ± 0.0035 |
| V (Å^3^) | 47.55 ± 0.049 | 47.44 ± 0.062 | 47.70 ± 0.017 | 47.56 ± 0.121 | | 47.46 ± 0.089 |
| ⟨D⟩_XRD-Scherrer_ (nm) | 25 ± 3 | 26 ± 2 | 25 ± 2 | 23 ± 2 | | 21 ± 1 |
| ⟨D⟩_XRD-W-H_ (nm) | 50 ± 1 | 21 ± 1 | 20 ± 2 | 18 ± 2 | | 17 ± 2 |
| Strain | 15×10^-4^ ± 3×10^-5^ | 7×10^-4^ ± 3×10^-5^ | 8×10^-4^ ± 3×10^-5^ | 8×10^-4^ ± 3×10^-5^ | | 9×10^-4^ ± 4×10^-5^ |
| ⟨D⟩_SEM_ (nm) ± σ_D_(nm) | 66 ± 12 | 55 ± 11.6 | 61 ± 13.4 | 58 ± 9.6 | | 61 ± 13.3 |

**Table S2.** EDX (atomic precent) of ZnO NPs with different Ag doping.

| **Element** | **ZnO** | **ZnO@Ag2%** | | **ZnO@Ag4%** | | **ZnO@Ag6%** | | **ZnO@Ag8%** | |  |
| --- | --- | --- | --- | --- | --- | --- | --- | --- | --- | --- |
| Zn | 47.85 | | 45.23 | | 30.41 | | 38.29 | | 44.22 | |
| O | 39.87 | | 44.28 | | 59.37 | | 38.54 | | 36.77 | |
| Ag | - | | 0.14 | | 0.32 | | 0.34 | | 0.71 | |
| C | 12.28 | | 10.35 | | 9.89 | | 22.83 | | 18.30 | |

**References**

1. Acharya, T. R. *et al.* Influence of nanoparticle size on the characterization of ZnO thin films for formaldehyde sensing at room temperature. *Sens. Actuators A: Phys.*, 114175 (2023).

2. Agarwal, S. *et al.* An efficient hydrogen gas sensor based on hierarchical Ag/ZnO hollow microstructures. *Sens. Actuators B: Chem.* **346**, 130510 (2021).

3. Barin, Ö. *et al.* Pivotal role of nucleation layers in the hydrothermally-assisted growth of ZnO and its H_2_ gas sensing performance. *Sens. Actuators B: Chem.* **371**, 132499 (2022).
